# Antilisterial Effect of* Rosa damascena* and* Nymphaea alba* in* Mus musculus*

**DOI:** 10.1155/2018/4543723

**Published:** 2018-01-23

**Authors:** Rida Batool, Asma Kalsoom, Iqra Akbar, Najma Arshad, Nazia Jamil

**Affiliations:** ^1^Department of Microbiology and Molecular Genetics, University of the Punjab, Quaid-e-Azam Campus, Lahore, Pakistan; ^2^Department of Zoology, University of the Punjab, Quaid-e-Azam Campus, Lahore, Pakistan

## Abstract

The present study was proposed to investigate the toxicological and prophylactic potential of ethanolic extracts of* Rosa damascena* and* Nymphaea alba* and their mixture in albino mice. For toxicity study, three different doses of plant extracts were orally administrated to three groups of mice for 14 successive days. Blood biochemistry and histological examinations of liver and kidney revealed that these extracts had no harmful effects up to 1000 mg/kg. To determine the prophylactic effects of* Rosa damascena*,* Nymphaea alba*, and their mixture, an infection model of* Listeria monocytogenes* was established in a pilot study. Establishment of infection was confirmed by changes in haematological parameters and reisolation of* Listeria monocytogenes* from different tissues. Results showed that these extracts alone or in combination could restrict the growth of* Listeria monocytogenes* in different organs. Neutrophils were high in positive control group but remained in normal range in all treated groups.* Listeria monocytogenes* was recovered in low numbers from animals treated with extract of single plant but was negligible in group treated with mixture of extract of plants. Platelets count was increased in treated groups as compared to control. Results confirmed that these extracts are potent source of antimicrobial compounds and that they have synergistic effect in combined form.

## 1. Introduction

From prehistoric times, herbs are being used as medicine for treatment of different illnesses [1]. About 80% of people living in developing countries use traditional medicines for therapeutic purposes [2, 3]. Medicinal plants are abundant source of bioactive compounds and are capable of reducing the infectious and noninfectious diseases; however, there are only few reports on their toxicity/side effects [4]. Human pathogens have been reported to become drug resistant even in developed countries. The situation is severe in underdeveloped countries because of uncontrolled usage of antibiotic and antibacterial products particularly in immune-compromised subjects [5]. The need of the time is to establish new and improved antimicrobial components to cope with the problem of emergence of resistant bacteria.

*Nymphaea alba* Linnaeus (family: Nymphaeaceae) is commonly known as water lily or water rose.* Nymphaea alba* (aquatic herb) consists of continuous shoots anchored with mud. This flora is generally found in ponds and lakes of warmer places of Africa, Europe, China, Southwest Asia, Russia, and India [6]. All the parts of plant were used by ancient people for treatment [7]. In traditional system of medication, all parts of this plant have been utilized to treat various illnesses [8]. Therapeutically,* Nymphaea alba* is used as aphrodisiac, astringent, cardiotonic, sedative, and anti-inflammatory [6]. It consists of various phytochemical compounds, for example, alkaloids, polyphenolic compounds, flavonoids, tannic acid, glycosides, gallic acid, and sterols. Most commonly it is used to cure gonorrhoea, diarrhea, and leucorrhoea because of antibacterial potential of this plant [9]. Various* in vivo* studies of* Nymphaea alba* reported that this plant has antidiabetic [10], antidiarrheal [11], hepatoprotective, antioxidant, and anti-inflammatory activities [12], intestinal *α*-glucosidase inhibition and toxicity [13], anxiolytic activity [14], and anticarcinogenic effect [8], whereas,* in vitro* studies also revealed antioxidant [15] and antibacterial activity [16].

*Rosa damascena* Miller is member of the genus* Rosa,* family Rosaceae [17, 18]. In ancient medicine,* Rosa damascena* has been utilized for many therapeutic effects such as curing chest and abdominal pain and digestion problems and reducing inflammation [19, 20]. The roots of this plant were used as cough remedy by North American Indian tribes [21]. Rosa oil has been reported as vapor therapy for curing various allergies and migraine [22]. Traditionally petals of* Rosa damascena* were reported for blood purifying properties [23]. It is a mild laxative [24]. It consists of antibacterial compounds such as citronellol, geraniol, and nerol. [25]. Rosa is used for curing depression, menstrual bleeding, skin problem, nervous stress, and headache in ancient medication [26]. In* Rosa damascena* petals flavonoids are present and are reported to strongly resist UV radiations (254 nm). Therefore, they are used in making of sunblock creams. Flavonoids also act as an antioxidant that protects the DNA from oxidative damage [19]. The flowers of* Rosa damascena* are reported to have antibacterial [25], anti-HIV [27], antioxidant [28], antidiabetic [29], and anti-inflammatory effects [30].

Both of these plants are used alone by herbal practitioners; however, their prophylactic potential has not been worked out so far. On the other hand, data available on the toxicological assessment of* Rosa damascena* and* Nymphaea alba* till 2000 mg/kg body weight has been studied separately, but no such information is available about their mixture to our knowledge [8, 23]. This information is required to enhance the confidence of their safety to human. Present study was planned to estimate the toxicological and prophylactic potential of* Rosa damascena*,* Nymphaea alba*, and their mixture in mammalian model.

## 2. Material and Methods

### 2.1. Plant Extract Preparation

Flowers of* Rosa damascena *(Gul-e-Surkh) and* Nymphaea alba* (Gul-e-Nilofer) were collected from Punjab, Pakistan, and were identified by Professor Dr. Sikandar Sultan (Department of Microbiology and Molecular Genetics, University of the Punjab, Lahore).* Rosa damascena* and* Nymphaea alba* were placed with voucher nos. ZD-IM-30 and ZD-IM-31 in Microbiology and Immunology Laboratory (Department of Zoology, University of the Punjab). Flowers were thoroughly washed with tap water, dried at room temperature under shade, and crushed into powder. Crushed flowers were extracted with absolute ethanol. The resulting extract was filtered and concentrated in rotary evaporator at 60°C.

### 2.2. Animals Housing and Feeding

Healthy male albino mice* (Mus musculus)*, 10–12 week old, weighing 30 ± 2 g were purchased from University of Veterinary and Animal Sciences, Lahore. They were kept in well-ventilated environment at temperature 28°C and 65% humidity. Animals had free access to standard pellet diet and water ad libitum. Mice were allowed to become acclimatized for 10 days prior to the experiment.

### 2.3. Toxicity Study

#### 2.3.1. Dose Preparation and Administration

Extracts of* Rosa damascena* and* Nymphaea alba* were mixed in equal ratio. Three doses, 500 mg/kg, 1000 mg/kg, and 1500 mg/kg body weight, were selected for* in vivo* toxicity testing. Plants extracts were mixed in normal flour. Pellets were made by mixing small quantity of water, dried under shade, and stored at room temperature. Animals were divided into four groups each consisting of ten animals by random sampling technique and labeled as G1 (500 mg/kg body weight), G2 (1000 mg/kg body weight), G3 (1500 mg/kg body weight), and C (control), respectively. After acclimatization period, the acute and chronic toxicity study was performed as per the OECD-423 guidelines [31]. Animals of G1, G2, and G3 were fasted for four hours every day, after which food pellets containing plant extracts were offered till complete consumption of the selected dose. Later on, animals were shifted on normal diet ad libitum. Control group was treated in similar way but after fasting it was fed on plain flour pellets.

#### 2.3.2. Physical Observation and Body Weight Measurement

Mice were observed regularly for behavioral changes such as abdominal constriction, hyperactivity, sedation, grooming, and mortality. Initial body weight of all groups of mice was recorded once before the beginning of the first oral administration, and final body weight of all groups was recorded at the time of termination of experiment.

### 2.4. Blood and Tissue Sampling

At days seven and fourteen after treatment, 5 mice from each group were randomly selected for bleeding. The animals were anesthetized with chloroform and the blood samples were collected from the heart in vacutainers without anticoagulant. The blood samples were allowed to clot for 15 minutes at room temperature. Serum was separated by centrifuging the blood at 3000 rpm for 10 minutes and stored at −20°C till further analysis. Small pieces of liver and kidney were collected with the help of sharp scissors and forceps. Tissues were preserved in 10% formalin and processed for histological examination. Tissue samples of liver and kidney were stained and thin sections were prepared according to Marco et al. (1992) [32]. Tissue sections were observed under light microscope at 1000x.

### 2.5. Toxicity Examination

Animals were observed daily for signs of toxicity including anxiety, cannibalism, depression, and weight loss, while the serum samples were subjected for evaluation of alkaline phosphate, alanine transaminase (ALT), bilirubin, uric acid, urea, and creatinine using autoanalyzer.

### 2.6. Infection Model

#### 2.6.1. Pilot Study

Pilot study was conducted to define the infective dose of the pathogen. For that purpose, animals (15) were divided into three groups, A, B, and C. Five ml of autoclaved nutrient broth was inoculated with* Listeria monocytogenes* and incubated at 37°C for 24 hours. After incubation, culture absorbance was adjusted to OD 1.00 ± 0.02 at 600 nm and CFU/ml were estimated, and two doses corresponding to 10^5^ and 10^7^ CFU/ml were prepared in normal saline. Groups A and B were exposed to 200 *μ*l containing doses of 10^5^ and 10^7^ CFU/ml, respectively. Four* Mus musculus* mice weighing 28 g were randomly selected and injected with 100 *μ*l and 200 *μ*l of the pathogen. Mice were observed regularly for change in behavior, eye secretions, sunken eyes, circumflex backbone, hyperactivity, sedation, and mortality. The weights were taken on daily bases. On day 3 after infection, animals were anesthetized with chloroform. Their skin was swabbed with absolute ethanol and dissected via properly sterilized sharp scissors and forceps. Impression of the organs (blood, heart, liver, spleen, and kidney) was taken on tryptic soya agar plates and incubated at 37°C for 24 hours.

### 2.7. Prophylactic Effects of Extracts on Progress of* Listeria monocytogenes* Infection

#### 2.7.1. Animals and Housing Conditions

Animals housing has been described in the previous section (Toxicity). Twenty-five* Mus musculus* mice were divided into five groups G1 (positive control), G2 (negative control), G3* (Rosa damascena)*, G4* (Nymphaea alba)*, and G5* (Rosa damascena* +* Nymphaea alba)*, respectively.

*(1) Dose Preparation and Administration*. Single doses (1000 mg/kg body weight) of* Rosa damascena* and* Nymphaea alba* extract, separately and in combined form (equal ratio), were mixed with flour; pellets were made, dried under shade, and stored at room temperature as it was mentioned before. One week before exposure to* Listeria monocytogenes* animals of G3, G4, and G5 were fed daily for 3 hours on diet containing extracts of medicinal plants till complete consumption of the selected dose and later on shifted to normal diet ad libitum. Animals of G1 and G2 were treated similarly; however, G1 and G2 received plain flour in the same quantity. At day 6, animals of G1, G3, G4, and G5 were exposed to 10^7^ CFU/ml of* Listeria monocytogenes* in 200*μ*l of saline orally, while G1 received the same quantity of normal saline.

### 2.8. Body Weight Measurement and Physical Observation

Mice of each group were tagged. Body weight of each mouse was taken regularly till end of experiment. Mice were observed regularly for behavioral changes such as sluggishness, circumflex backbone, sunken eyes, ruffled fur, abdominal constriction, and mortality.

### 2.9. Blood Sampling

Following 7 days of infection, five mice from each group were sacrificed. The animals were anesthetized with chloroform and the blood samples were collected from the heart in vacutainers with anticoagulant. The blood samples were kept in refrigerator till further analysis. Samples were sent to BioMed clinical laboratories (Lahore, Pakistan) where they were processed manually for the hematological analysis. The following hematological parameters were selected: total RBC (red blood cell), hemoglobin, HCT (hematocrit), MCV (mean corpuscular volume), MCHC (mean corpuscular hemoglobin concentration), platelets, WBC (white blood cell), neutrophils, lymphocytes, and monocytes.

### 2.10. Reisolation of Bacteria

With the help of sterile scissors and forceps, impressions of liver, kidney, spleen, heart, and blood of each animal were taken on LB-agar plates and incubated at 37°C for 24 hours. Small pieces of the liver, kidney, and spleen of the animals were taken into eppendorf containing 1 ml of distilled, autoclaved water. The organs were triturates in water with the help of sharp objects. From each eppendorf, 100 *μ*l was spread on the media plates and incubated at 37°C for 24 hours.

### 2.11. Statistical Analysis

Data was analyzed using one-way ANOVA followed by DMRT in SPSS software (version 16.0). Data are presented as mean ± SE and differences at *p* < 0.05 were considered statistically significant.

## 3. Results

### 3.1. Toxicity of Mixture of* Rosa damascena* and* Nymphaea alba*

#### 3.1.1. Physical Observations and Body Weight

The initial and final body weights of both treated and nontreated mice were comparable (Table 1). No signs and symptoms of toxicity including hyperactivity, sedation, and mortality could be observed in any treatment group.

#### 3.1.2. Effects of Extracts on Biochemical Parameters

The acute and chronic effects of the mixture of extracts of* Rosa damascena* and* Nymphaea alba* on biochemical parameters of blood are given (Table 2). A significant depletion in ALT was noticed after 14 days of experiment while all other parameters including alkaline phosphatase, bilirubin, blood urea, creatinine, and serum uric acid remained unaltered.

#### 3.1.3. Histopathological Observations

**Figure 1:**
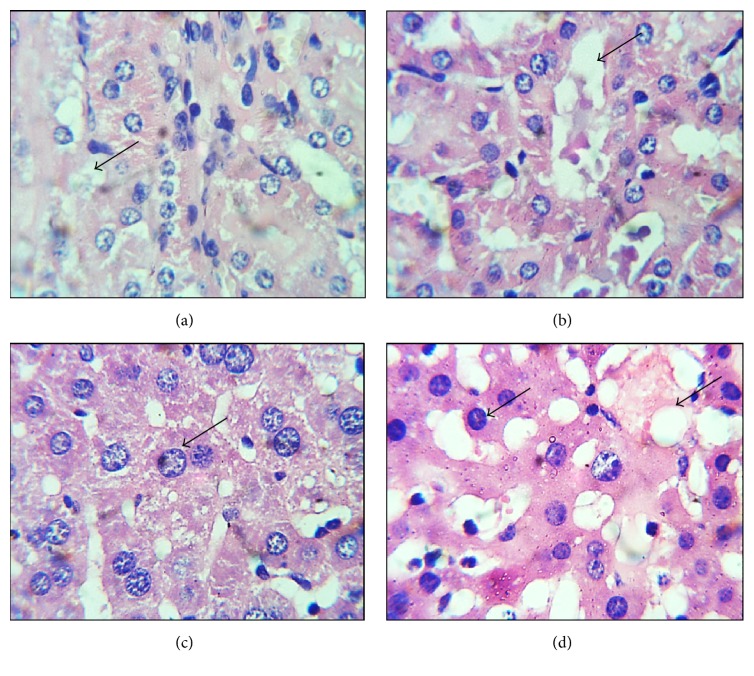
Comparison of photomicrographs of liver and kidney sections of animals exposed at 1500 mg/kg of mixture of extracts and control. (a) Control kidney section showed normal nucleus in lymphocytes and vacuoles are small in size. (b) Treated kidney showed slight abnormality in kidney, vacuole, and deformed nucleus can be observed. (c) Control liver section exhibited normal hepatocyte nuclei and intracellular spaces. (d) Treated liver revealed abnormalities in hepatocyte nucleus and large vacuoles indicated by arrows.

Histopathological effects of mixture of ethanolic extracts of* Rosa damascena* and* Nymphaea alba* on liver and kidney sections were compared with the control group. Microscopic observation showed that there was no difference in the liver and kidney sections between the control group and mice treated with doses of 1000 mg/kg. However, at 1500 mg/kg animals exhibited some signs of toxicity. Toxicity was observed at tissue level but it could not be noticed in blood biochemistry (Figure 1).

### 3.2. Infection Model

#### 3.2.1. Pilot Study: Setting Dose of* Listeria monocytogenes*

*Listeria monocytogenes* induced the infection within 2 days. Animals shed their weight from 28 g to 21 g. Typical symptoms of* Listeria monocytogenes* in mice include sluggishness, eye color becoming dark red, and fur starting to ruffle, with circumflex backbone being the clear sign and symptom (Table 3). Pathogen* (Listeria monocytogenes) *was reisolated from the heart, liver, blood, and lung. On impression plates, there were too numerous colonies to count which was the indication for the spread of infection throughout the body.

### 3.3. Main Study: Effects of Extracts on* Listeria monocytogenes* Infection in Mice

#### 3.3.1. Physical Observation and Body Weight Measurement

Animals became slightly sluggish after two days of infection as compared to negative control. One animal of group three (G3) died while another animal of this group suffered from circumflex back bone. Minor variations in the body weights of the mice of infected groups (G1, G3, G4, and G5) were observed (*p* > 0.05). However, when comparing the body weight between the treated groups and the negative control, it was found that animals of G2 (negative control) gained weight (Table 4).

#### 3.3.2. Reisolation of* Listeria monocytogenes* from Tissues

Impressions of the organs (liver, kidney, spleen, heart, and blood) of each animal were taken on tryptic soya agar plates and incubated at 37°C for 24 hours. There were too numerous colonies on the plates indicating that infection had been reached to organs. Pathogen was reisolated from the selected organs but the number of colonies was few. In group G3 (treated with* Rosa damascena*), reisolation of pathogen from liver tissues was 2/4, lung 1/4, kidney 0/4, heart 1/4, and spleen 0/4. In case of group G4 (treated with* Nymphaea alba*) the* Listeria monocytogenes *could be reisolated from only one animal but* Listeria monocytogenes *could be reisolated from all organs including liver, lung, kidney, heart, and spleen (1/5). However, in group treated with mixture, few colonies of* Listeria monocytogenes *were isolated from liver and heart of only one animal (1/5) (Table 5).

### 3.4. Hematology

Two hematological parameters, neutrophils and lymphocytes counts, were found sensitive to* Listeria monocytogenes* infection. Although no significant variations in RBC, WBC, Hb, and PCV hematological indices could be observed, ratio of neutrophils and lymphocytes altered in* Listeria monocytogenes *infection. A significant reduction in lymphocytes and elevation in neutrophil were observed in positive control group (G1) compared to negative control (G2). Reversion of these parameters to level of negative control was noticed in all treated groups (Table 6). Moreover, platelet counts in all treated groups were found to be elevated.

## 4. Discussion

Emergence of resistance in pathogenic bacteria is being reported from all parts of the world, which has accelerated research in the field of new therapeutic agents from different resources including medicinal plants. A number of plant species have been exploited for medicinal purposes with variable success. Medicinal plants and their extracts are generally considered safe and effective. However,* in vivo* evidence of their safety and effectiveness is mandatory and has been less documented. Shohayeb et al. (2014) [33] in his* in vitro* studies on* Rosa damascena* reported its antimicrobial and antioxidant activities. Similarly,* Nymphaea alba *has been used in ancient times as an antiseptic, radical scavenger and astringent, while rhizomes were applied externally as a rubefacient [12, 34], and use of this herbal medicine is increasing day by day [35]. Current study is designed to evaluate the* in vivo* antibacterial effects of mixture of* Rosa damascena* and* Nymphaea alba *against* Listeria monocytogenes *infection.

During the screening of drugs, determination of LD_50_ is a first step for the evaluation and assessment of toxicity of a substance. This is the preliminary screening step that has to be performed with all compounds for initial evaluation of toxic manifestation. Data obtained from acute toxicity experiments may serve for dose determination in animal studies [35]. Previous studies reported no toxicity of both plants till 2000 mg/kg [14, 36], but for mixtures no reports were available. Therefore, in this study, three doses (500 mg/kg, 1000 mg/kg, and 1500 mg/kg) of extracts of two medicinal plants (*Rosa damascena* and* Nymphaea alba*) in combination (1 : 1) were subjected to* in vivo* toxicity and other side effects.

None of the doses had adverse effects on the behavioral response of subjects except 1500 mg/kg up to 15 days of continued treatment. There was no morbidity or mortality nor effect on body weights of mice at 1000 mg/kg dose of both extracts. These findings are inconsistence with Bose et al. (2012) [11] who reported similar results up to 2000 mg/kg body weight for each of plant extracts alone. The LFT, RFT, and liver and kidney histology are reliable markers of toxicity [11]. All the biochemical parameters were within normal range except ALT (alanine amino transaminase).

Alanine amino transaminase acts as a catalyst in the formation of oxaloacetic and pyruvic acid by transferring *α*-amino groups from aspartate and alanine to the *α*-keto group of ketoglutaric acid, respectively. These chemical compounds play important role in citric acid cycle. If the ALT levels increase in serum, this can cause liver damage; however, low level of ALT in serum is not considered toxicity marker [37]. Treatment of mice at dose 1000 mg/kg did not cause any pathologic damage to the liver and kidney. However, slight necrosis and vacuolation in liver and kidney tissues were observed in animals treated with 1500 mg/kg of mixture. These finding are in consistence with previous studies on single plant extracts on microanatomy of kidney up to 2000 mg/kg BW [38, 39]. Our studies indicate that the plants are safe individually till 2000 mg/kg BW, but their combination could cause necrosis in the tissues at 1500 mg/kg body weight.

A number of reports are available on the inhibitory activity of medicinal plants and different phytochemicals against pathogenic and nonpathogenic organisms through* in vitro* procedures [19, 40, 41]. However, all these results need to be validated in* in vivo* settings [42]. For* in vivo* investigations, infection models had to be established. We used* Listeria monocytogenes* infection model in which changes in behavior, eye secretions, sunken eyes, circumflex backbone, hyperactivity, sedation, and mortality were important signs of infection. Results of pilot studies clearly indicated that intraperitoneal inoculation of 200 *μ*l of 10^7^ CFU/ml suspension of* Listeria monocytogenes *could successfully establish systemic infections.

The extract of* Rosa damascena* and* Nymphaea alba *restricted the growth of pathogens* in vitro*. These findings were further validated by* in vivo* system. Hematological parameters were recorded to validate antibacterial efficacy of extracts. It is also used to explain the effects of blood relating functions of a plant extract or its products [43].

The potential of extracts in reducing infection was also monitored while recording physical signs of disease including change in behavior, eye secretions, sunken eyes, circumflex backbone, hyperactivity, sedation, and mortality in infected mice. High count of neutrophils and low count of lymphocyte or vice versa are indicators of different diseases and disorders. They act as primary defenders against bacterial infection physiological stress. The important components of immune system are lymphocytes, which are type of white blood cells. The difference between lymphocytes and neutrophils in adult animals is quite different: human blood is neutrophil rich (50–70% neutrophils, 30–50% lymphocytes), whereas mouse blood has a strong preponderance of lymphocytes (75–90% lymphocytes, 10–25% neutrophils) [44]. An increased need for neutrophils, as with an acute bacterial infection, will cause an increase in total number of neutrophils. The TLC, ESR, and DLC are reliable indicators of bacterial infection [45].

The DLC of G3 and G5 revealed a reduction in number of the neutrophils to lymphocyte ratio as compared to G4. The neutrophil to lymphocyte ratio was high in G1 as compared to G2, which indicated the presence of infection at tissue level. An increase in one type of white blood cells can cause a decrease in other types of white blood cells. For example, in bacterial infection, neutrophils count increases and lymphocytes count decreases. Similar results have been demonstrated by Dolma et al. (2014) [46] indicating the increase production of neutrophils in animals during an infection with decrease in lymphocytes. These leukocytes have generally been thought to be important primarily for combating infection with extracellular bacterial pathogens. However, recent publications clearly indicate that neutrophils are crucial for restricting the growth of* Listeria monocytogenes* in mice [47].

In culmination, the hematological analysis of* in vivo* antibacterial activity in* Mus musculus* against* Listeria monocytogenes* provided clear indication that extracts were helpful in restricting the infection. The infection led to elevation in number of lymphocytes and neutrophils, but pretreatment with* Rosa damascena* and* Nymphaea alba* and their mixture could successfully restrict the infection, keep the number of lymphocytes, neutrophils (*p* value 0.001), and WBCs in normal range, and enhance the production of platelets.

These findings were further strengthened by recording reisolation of pathogen from tissue on LB-agar plates. The results showed that infection spreads throughout the body in positive control group. However, the numbers of the colonies reisolated from the treated groups (G3 and G5) were less than those from G1 (positive control). This further indicates that the plants play some role in reducing infection. G4* (Nymphaea alba)* alone failed in controlling the infection; however, in G5 (the group treated with mixture of extracts), the reduction was significant. It could be due to the synergistic effects of both extracts. The findings indicate that* Rosa damascena* and its mixture with* Nymphaea alba* help in controlling infection, but* Nymphaea alba* alone cannot control spread if infection is* in vivo*, highlighting the need for* in vivo* validation of* in vitro* findings.

Platelets are type of cells that recognize damaged blood vessels and help in binging of these vessels. They are not directly associated with the bacterial infections [31]. To our surprise high levels of platelets were observed in animals treated with mixture of plant extracts, indicting the role of these extracts in thrombocytogenesis. This aspect needs further confirmation, which may lead to further application of these extracts in other diseases like dengue fever.

## 5. Conclusion

The* in vivo* antibacterial evaluation of selected medicinal plant extracts indicated that they have the potential to combat bacterial infection. Neutrophils count increases during infection; however,* Rosa damascena* and mixture of extracts could successfully restrict spread of pathogen, which was supported by normal counts of leucocytes, neutrophils, and lymphocytes. The mixture of extracts showed some abnormalities in tissues at dose of 1500 mg/kg body weight. Future studies are needed for characterization of the active component present in these plants.

## Figures and Tables

**Table 1:** Effects of mixture of extracts on the body weight (g) of *Mus musculus*.

Groups	Treatment (mg/kg)	Initial weight	Final weight	Weight change
Control	0	29.6 ± 1.25	31.20 ± 1.76	1.6 ± 1.72
Group 1	500	31.82 ± 2.13	33.05 ± 2.12	1.23 ± 2.02
Group 2	1000	30.03 ± 2.77	31.45 ± 1.98	1.42 ± 2.14
Group 3	1500	32.05 ± 2.97	33.29 ± 2.10	1.24 ± 1.95

Comparison among different treatment groups was performed using one-way ANOVA. No significant differences in body weight of different treatment groups could be observed. Data are presented as mean ± SEM.

**Table 2:** Effects of extracts on biochemical parameters.

Parameters	Groups	
	Control (*n* = 8)	Treated (*n* = 8)
Mortality	0	0
Morbidity	0	0
Alanine transaminase (U/L)	83.3 ± 1.32	29.67 ± 0.333^*∗*^
Alkaline phosphatase (U/L)	426 ± 49.7	333.0 ± 57.501
Bilirubin (mg/dl)	0.438 ± 0.19	0.433 ± 0.066
Blood urea (mg/dl)	40.3 ± 3.73	35.00 ± 3.152
Creatinine (mg/dl)	0.64 ± 0.18	0.500 ± 0.0577
Serum uric acid (mg/dl)	4.24 ± 0.73	2.533 ± 0.392

Data are presented as mean ± SEM. Data were analyzed using independent sample *t*-test. Asterisk (in row) shows significant difference with control group at *p* ≤ 0.05.

**Table 3:** Effects of different doses on mice.

	Group A (100 *µ*l)	Group B (200 *µ*l)	Group C (control)
*Parameters*			
Mortality	−	−	−
Change in behavior	±	++	−
Eye swelling	−	++	−
Discharge from eye	−	+	−
Shape of neck (circumflex backbone)	−	++	−
Sedation	−	+	−
Movement by forelimbs	−	++	−
Hyperactivity	−	++	−
*Reisolation*			
Blood	+	+++	−
Liver	+	+++	−
Lung	+	++	−
Kidney	−	+	−
Spleen	−	++	−
Heart	+	++	−

**−**: nil; ±: slight; +: few; ++: moderate; +++: high.

**Table 4:** Effects of ethanolic extracts of *Rosa damascena*, *Nymphaea alba*,and their mixtures on the body weight of mice treated at dose 1500 mg/kg.

Groups	Treatment	Average weights				
		Day 1	Day 2	Day 3	Day 4	Day 5
Group 1	BI	30.4 ± 0.65	32 ± 0.58	32 ± 0.4	28.8 ± 0.52	32.6 ± 0.72
Group 2	NT	31.6 ± 0.57	35 ± 0.28	35 ± 0.28	35 ± 0.37	34.6 ± 0.20
Group 3	BI + R	30 ± 0.45	29 ± 0.67	29 ± 0.85	30 ± 0.41	30 ± 0.55
Group 4	BI + N	30.8 ± 0.5	33.4 ± 0.64	33.4 ± 0.58	29.4 ± 0.58	32.6 ± 0.46
Group 5	BI + N + R	27.8 ± 0.52	32 ± 0.28	29.4 ± 0.33	30.4 ± 0.33	32.6 ± 0.5

NT: no treatment, BI: bacterial infection, N + R: *Nymphaea alba* + *Rosa damascena*, N: *Nymphaea alba*, R: *Rosa damascena*. Data are presented as mean ± S.E.M. Data are analysed using one-way ANOVA. No significant differences could be recorded among groups throughout experiment.

**Table 5:** Reisolation of *Listeria monocytogenes* from different organs.

	Groups				
	Positive control (*n* = 5)	Negative control (*n* = 5)	*Rosa damascena *(*n* = 5)	*Nymphaea alba *(*n* = 5)	*Rosa + Nymphaea *(*n* = 5)
*Parameters*					
Morbidity	5/5	0/5	4/5	2/5	1/5
Mortality	0/5	0/5	1/5	0/5	0/5
*Reisolation*					
Liver	5/5	0/5	2/4	1/5	1/5
Lung	5/5	0/5	1/4	1/5	0/5
Kidney	5/5	0/5	0/4	1/5	0/5
Heart	5/5	0/5	1/4	1/5	1/5
Spleen	5/5	0/5	0/4	1/5	0/5

**Table 6:** Effects of extracts separately and in combination on haematological parameters.

Parameters	Groups					*p* value
	Positive control	Negative control	*Rosa damascena*	*Nymphaea alba*	*Rosa + Nymphaea*	
RBCs (×10/*µ*l)	7.76 ± 0.30	7.77 ± 0.34	8.07 ± 0.26	6.65 ± 1.13	8.28 ± 0.28	0.338
Hemoglobin (g/dl)	12.02 ± 0.55	11.6 ± 0.72	12.4 ± 0.77	10.35 ± 1.69	13.2 ± 0.31	0.420
PCV (%)	39.65 ± 1.68	40.16 ± 3.53	42.3 ± 4.01	33.27 ± 5.17	43.8 ± 1.81	0.259
MCV (fL)	53.0 ± 1.15	51.7 ± 2.62	57.8 ± 0.66	50.42 ± 1.31	54.6 ± 2.22	0.090
MCH (%)	16.85 ± 0.85	15.08 ± 0.38	15.4 ± 0.44	15.72 ± 0.56	17.1 ± 1.15	0.149
MCHC (%)	30.04 ± 0.67	29.28 ± 0.79	29.4 ± 0.97	30.97 ± 0.44	28.9 ± 1.16	0.503
Platelets (×10/*µ*l)	238 ± 22.53^b^	247 ± 81.47^b^	435 ± 19.15^b^	473 ± 136.64^ab^	597 ± 30.96^a^	0.013
WBCs (×10/*µ*l)	6.02 ± 0.43	6.32 ± 1.37	3.96 ± 0.09	5.45 ± 1.25	3.8 ± 0.65	0.142
Neutrophils (%)	27.50 ± 2.5^a^	12.60 ± 1.07^b^	14.6 ± 0.70^b^	13.25 ± 2.68^b^	15.4 ± 3.53^b^	0.001
Lymphocytes (%)	62.5 ± 4.78^b^	78.6 ± 5.03^a^	83 ± 1.22^a^	75 ± 4.56^ab^	79 ± 5.15^a^	0.039
Monocytes (%)	5.25 ± 4.09	5 ± 2.7	3.6 ± 1.31	4.0 ± 2.4	4.3 ± 2.02	0.230
Eosinophils (%)	5.0 ± 1.5	5.0 ± 1.0	4.1 ± 0.70	3.75 ± 0.47	5.5 ± 0.55	0.195

Data are presented as mean ± SEM; different alphabets (in a row) indicate significant variation among groups at *p* ≤ 0.05. The variables having no superscript letters indicate no significant difference among groups.
